# Catch Data Can Unravel Elasmobranch Aggregation Dynamics and Group Behaviours

**DOI:** 10.1002/ece3.71107

**Published:** 2025-03-30

**Authors:** A. G. McInturf, M. Cantor, I. A. Bouyoucos, T. K. Chapple, S. F. Debaere, K. Eustache, J. Mourier, S. Planes, J. A. Sulikowski, K. W. Zillig, N. A. Fangue, J. L. Rummer

**Affiliations:** ^1^ Coastal Oregon Marine Experiment Station Oregon State University Newport Oregon USA; ^2^ Department of Fisheries, Wildlife and Conservation Sciences Oregon State University Corvallis Oregon USA; ^3^ Marine Mammal Institute Oregon State University Newport Oregon USA; ^4^ Australian Research Council Centre of Excellence for Coral Reef Studies James Cook University Townsville Queensland Australia; ^5^ PSL Research University, EPHE‐UPVD‐CNRS, USR 3278 CRIOBE Université de Perpignan Perpignan France; ^6^ ECOSPHERE, Department of Biology, University of Antwerp Antwerp Belgium; ^7^ Marine Biology, College of Science and Engineering James Cook University Townsville Queensland Australia; ^8^ Institute for Biodiversity and Ecosystem Dynamics University of Amsterdam Amsterdam the Netherlands; ^9^ MARBEC University of Montpellier, CNRS, IFREMER, IRD Sète France; ^10^ Wildlife, Fish and Conservation Biology Department University of California Davis California USA; ^11^ Fisheries, Wildlife and Conservation Biology University of Minnesota St. Paul Minnesota USA

**Keywords:** animal behaviour, fisheries, interactions, marine conservation, sociality

## Abstract

Elasmobranchs (i.e., sharks, skates, rays), known for their cognitive abilities and complex behaviours, often form aggregations that are thought to be crucial for their survival and evolutionary success. However, understanding the drivers behind these aggregations remains challenging due to the dynamism of the marine environment and the difficulty of observing these species directly. Here, we aim to address these challenges by introducing a methodological framework for analysing catch data to infer aggregation behaviour. Within this framework, we outline key metrics to explore, such as the number and density of individuals captured, phenotypic traits, drivers of co‐occurrence, individual identification, and kin structure. We then demonstrate how to use this framework in a case study of juvenile blacktip reef sharks ( *Carcharhinus melanopterus* ) in Moorea, French Polynesia, to determine its real‐world application and identify potential limitations. Our results reveal that juvenile blacktip reef sharks around Moorea tend to aggregate during early life stages and that these aggregations appear non‐social, indicative of environmental rather than social drivers. We also find that, while catch data can provide valuable insights into elasmobranch aggregations, they must be complemented with targeted research methods to maximise the available data advised within our framework. As findings from our case study demonstrate, this framework has the capacity to broaden our knowledge of elasmobranch aggregations and social behaviours, underscoring the importance of dedicated efforts in research and conservation to manage these vulnerable species effectively.

## Graphical abstract

In this study, we introduce a methodological framework for analyzing catch data to infer aggregation behavior in elasmobranchs. We then demonstrate how to use this framework in a case study of juvenile blacktip reef sharks ( *Carcharhinus melanopterus* ) in Moorea, French Polynesia, to determine its real‐world application and identify potential limitations.

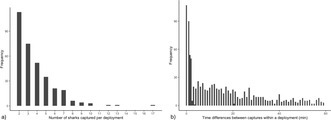

## 1 Introduction

Animal aggregation formation can have substantial effects on evolutionary and ecological processes as a widespread strategy across taxa (e.g., Krause and Ruxton 2002; Kurvers et al. 2014; Papastamatiou et al. 2020). Generally thought to confer some fitness advantage, aggregations can vary widely in form and purpose, based on intrinsic individual or species traits and environmental conditions (Hofmann et al. 2014). A prime example is aggregations of Chondrichthyans, a group of organisms that has existed for ~450 million years and is comprised of roughly 1200 extant, highly diverse species, varying in size, diet, reproductive strategy, physiology, and habitat. Within this class, aggregations have been observed across several pelagic, coastal, and reef species, predominantly within subclass Elasmobranchii (sharks, skates, and rays; e.g., grey reef [ *Carcharhinus amblyrhynchos* ; Papastamatiou et al. 2020], scalloped hammerhead [ *Sphyrna lewini* ; Klimley and Nelson 1984], blacktip reef [ *Carcharhinus melanopterus* ; Mourier et al. 2012; Mourier et al. 2017a; Mourier and Planes 2021], leopard [ *Triakis semifasciata* ; Hight and Lowe 2007; Nosal et al. 2013, 2014], and basking [ *Cetorhinus maximus* ; Sims et al. 2000] sharks). These aggregations take many forms and are driven by both non‐social and social mechanisms (i.e., environmental features and resources or the presence of conspecifics, respectively; McInturf et al. 2023). While feeding aggregations are common (e.g., whale sharks [ *Rhincodon typus* ], Hoffmayer et al. 2007; manta rays [*Mobula* spp.], Armstrong et al. 2016), more complex behaviors have also been reported. For example, basking sharks will often gather in plankton‐rich habitats to feed but will also seasonally cease feeding and circle in large “torus” formations, presumably for courtship (Sims et al., 2022). Other species demonstrate sex‐specific behavioral strategies in which females will aggregate in specific habitats to avoid males (e.g., small‐spotted catsharks [*Scyliorhinus canicula*]; Jacoby et al., 2010), or to behaviorally thermoregulate for gestation (i.e., maternal thermophily; round stingrays [ *Urobatis halleri* ]; Jirik & Lowe, 2012). More structured associations, such as specific social groupings in which co‐occurrence is driven by individual identity, have also been documented (e.g., Wobbegong sharks [ *Orectolobus maculatus* ]; Armansin et al. 2016). Collectively, these demonstrate the complexity and diversity of aggregation behaviors across elasmobranchs and underscore the need for further investigation into their ecological and evolutionary drivers.

Indeed, the widespread nature of aggregation formation across several elasmobranch orders suggests that they serve important evolutionary functions worthy of further examination (see Jacoby et al. 2012 and Papastamatiou et al. 2022 for review). Yet, to date, robust analyses of the mechanisms underlying aggregation formation in elasmobranchs have been relatively restricted to a few species (e.g., Armansin et al. 2016; Guttridge et al. 2011; Mourier and Planes 2021; Papastamatiou et al. 2020; Perryman et al. 2019). This is largely due to logistical constraints, which have historically made it challenging to observe elasmobranch behaviour and identify the drivers of individual co‐occurrence. Despite recent technological advancements (e.g., Guttridge et al. 2010; Haulsee et al. 2016; Jacoby and Freeman 2016; McCauley et al. 2016), measuring individual interactions in the field remains a formidable challenge for most species (Finucci et al. 2018; Mourier et al. 2012), largely due to inconsistent access to animals and the difficulties associated with deploying devices to collect behavioural data (e.g., mobile transceivers, animal‐borne cameras; Barkley et al. 2020; Haulsee et al. 2016). Current technology is particularly limiting for small species or individuals at early life stages, when they may be unable to bear most commercially available tags. Moreover, comprehensive monitoring over relevant timescales is both time‐demanding and costly.

A potential solution for obtaining more consistent information on elasmobranch interactions involves leveraging catch data, typically derived from two primary sources: research/monitoring activities or fisheries (i.e., commercial, subsistence, or recreational). Although catch data are primarily collected for purposes such as assessing population demographics or distribution patterns, some studies have already inferred potential aggregations or spatial segregation based on the number, timing, and demographics of sharks captured (e.g., Elisio et al. 2016; Flammang et al. 2011; Mucientes et al. 2009). However, few studies have specifically aimed to discern whether the species captured are engaging in social grouping or whether they are non‐socially aggregating (but see Finucci et al. 2018). This is likely because catch datasets vary in breadth of information (i.e., on traits such as size, sex, genetic relatedness, species identification, etc.) and spatiotemporal scale, and datasets often lack the comprehensive detail necessary to identify the drivers underpinning aggregations, whether they be environmental conditions, resources, or conspecifics.

Despite these potential obstacles, catch data could still provide valuable insights into the social and ecological dynamics of elasmobranch aggregations when complemented with more targeted research methods. For instance, these data could be used to identify the environmental contexts where social interactions are likely to occur, such as in certain locations or habitats. This approach echoes the early stages of avian population and social ecology research, where patterns of individual capture within the same population over multiple generations were observed using nest boxes and mist nets. These efforts inspired progressively detailed studies on social behavior and social learning in passerine birds (*Paridae*; e.g., Aplin et al. 2013, 2015; Farine et al. 2012; Lack 1964; Sheldon et al. 2022). Similarly, catch data can facilitate the exploration of potential social context in elasmobranch species or individuals that are challenging to access, tag, and/or observe consistently. For example, such data have revealed the apparent formation of aggregations in deep‐sea Chondrichthyan species (Finucci et al. 2018). This method can also be particularly useful for studying life stages where traditional tagging methods are impractical. Specifically, catch records from research surveys can monitor the occurrence of neonates or juveniles that may be too small for acoustic tags (depending on the species) and/or occupy habitats that limit the efficacy of monitoring (i.e., shallow waters).

Yet, due to limited exploration of methods for integrating catch data into socioecological studies, the potential and limitations of this approach remain unclear. Here we explore the extent to which catch data can be utilised to identify aggregations and differentiate between social groups and non‐social aggregations in elasmobranchs. We first present a broad framework for assessing these data to test common behavioral hypotheses (Table 1). We propose key metrics to explore, such as the number and density of individuals captured, phenotypic traits, drivers of co‐occurrence, individual identification, and kin structure, to confirm the presence of aggregations and identify their potential drivers. These metrics can be tailored to different study systems and research questions, with the goal of enriching the available body of knowledge on elasmobranch socioecological behaviors. We then present a case study analysing 10 years of catch data from juvenile blacktip reef sharks ( *Carcharhinus melanopterus* ) in Moorea, French Polynesia, to demonstrate the use of this framework. We conclude by discussing the assumptions and caveats of this approach and propose additional data collection methods and parameters to improve the use of catch data in socioecological research.

**TABLE 1:** Proposed descriptive metrics to help elucidate whether elasmobranch aggregations are occurring in catch data and whether these aggregations are likely to be social or non‐social (cf. McInturf et al. 2023). Hypotheses and predictions represent a non‐exhaustive list of generalized examples. Note that metrics and study outcomes may vary by species, life stage, spatial scale, and sampling period. Aggregation types may also not be mutually exclusive (McInturf et al. 2023). The asterisk (*) indicates data that are already commonly taken in fisheries.

Descriptive metric	Example hypotheses	Example predictions
i. Number and density of individuals captured*	H1: Individuals tend to *aggregate* rather than be solitary	H1P1: Elasmobranchs will be captured more often with other elasmobranchs than individually across (or within) capture sites/sets
ii. Characterisation of individual phenotypic traits*	H1: Individuals form *non‐social aggregations*	H1P1: Elasmobranchs will be consistently captured with other elasmobranchs, but individual traits will vary (*e.g*., size, sex)
	H2: Individuals form *non‐specific social groups*	H2P1: Elasmobranchs will be captured with individuals of similar traits (*e.g*., small individuals together, females together)
*iii. Evidence of drivers of co‐occurrence*	H1: Individuals form *non‐social aggregations*	H1P1: Elasmobranch co‐occurrence will correspond to a given environmental condition across (or within) sites/sets (*e.g*., temperature)
		H1P2: Elasmobranchs that co‐occur will consistently demonstrate evidence of feeding prior to capture (*e.g*., via stomach content analysis – excluding bait – or body condition) across (or within) sites/sets
	H2: Individuals form *social groups*	H2P1: Elasmobranchs will consistently co‐occur across (or within) sites/sets independently of variation in environmental conditions
*iv. Identification of specific individuals*	H1: Individuals form *non‐social aggregations*	H1/H2P1: Elasmobranchs will be consistently captured with other elasmobranchs, but individuals vary
	H2: Individuals form *non‐specific social groups*	
	H3: Individuals form *specific social groups*	H3P1: Elasmobranchs will be captured with the same individuals on multiple occasions
*v. Identification of kin structure*	H1: Individuals form *non‐social aggregations*	H1P1: Elasmobranchs will be consistently captured with other, unrelated elasmobranchs
	H2: Individuals form *non‐specific social groups*	H2P1: Elasmobranchs will be captured with individuals with whom they are genetically related
	H3: Individuals form *specific social groups*	H3P1: Elasmobranchs will be consistently captured with the same individuals with whom they are genetically related

## 2 Materials and Methods

### 2.1 A Framework for Using Catch Data to Explore Shark Aggregations

Establishing a framework for measuring interactions in elasmobranchs, whether social or non‐social, hinges on a clear definition of what constitutes an aggregation. We employ a recently proposed definition as “the co‐occurrence of two or more individuals in space and time due to the deliberate use of a common driver” (McInturf et al. 2023). Building on this definition, we propose that, by analysing up to five descriptive metrics within a set of elasmobranch catch data (Table 1), it is possible to discern whether aggregations are occurring and then infer whether these aggregations are likely to be social or non‐social in nature. In the following sections, we describe and outline specific predictions associated with each metric. Ideally, these metrics should be considered collectively for each set of data or across datasets for a given species. Additionally, while these metrics offer valuable guidance, researchers will also have to make informed decisions regarding the spatial scale and temporal sampling period that best identify potential aggregations, based on study question(s), system, and available data (McInturf et al. 2023). A comprehensive analysis of these metrics at the appropriate scales can then provide robust evidence to support (or not support) the need for further, more quantitative investigations into the aggregation behaviour of a given species.

#### 2.1.1 Number or Density of Individuals Captured

Calculating the number or density of individuals captured is foundational for inferring aggregation behaviour, regardless of whether it emerges from social or non‐social mechanisms. These data are commonly collected during any fishing or sampling effort. Depending on data availability and the spatiotemporal scale of interest, this metric needs to be examined within multiple sets or deployments to determine the degree of consistency in multiple versus solitary individuals captured. Though there has been some variation in the reported minimum number of individuals required to form an aggregation, a generalisable threshold is two or more (Allee 1927; Fouché et al. 2019; McInturf et al. 2023). Consequently, understanding the number (or density; Finucci et al. 2018) of animals captured within a sampling period is the first step to identifying aggregations and potential social groups. If aggregating is a common behaviour for the species or population, there should be a high prevalence of multi‐animal captures compared to individual captures across sampling periods. Ideally, sufficient data exist to determine whether individual animals are statistically more likely to be captured with other individuals than alone or by chance (e.g., Bejder et al. 1998). A non‐random capture pattern will support the hypothesis of aggregation and, considering individual traits, will warrant further investigation into the drivers of these aggregations (i.e., social or non‐social). Of note, there are cases in which number/density are reflective of other phenomena (e.g., mothers giving birth to multiple offspring in one location) or methodological approaches; for instance, the goal of targeted elasmobranch fishing may be to capture as many animals as possible per deployment, likely biasing our assessment of aggregation behaviour compared to fisheries or surveys in which elasmobranchs are captured incidentally. Additionally, depending on the scale of the fishing or survey effort, individuals may be captured sufficiently far apart in space and/or time that it may be unreasonable to assume they are aggregating. Thus, the value of this metric in examining aggregation potential will depend on obtaining sufficient contextual data on fishing or survey approaches. It is also necessary to supplement these data with those from other metrics to more robustly infer interindividual dynamics.

#### 2.1.2 Characterisation of Individual Phenotypic Traits

Though aggregations can be shaped by many mechanisms, the benefits incurred while aggregating can advance with the association of other similar individuals (i.e., social assortativity) (Mourier and Planes 2021). Thus, when multiple animals are captured, data on individual traits can provide further insight into the nature of potential aggregations. As above, these data are often recorded in fisheries (e.g., length) and research surveys (e.g., precaudal, total, and fork length, various girths, and sex), and can offer context for co‐occurrence. A specific size distribution within a capture set, for example, can indicate assortative grouping by age class (e.g., lemon sharks, *Negaprion brevirostris* ; Guttridge et al. 2011). Smaller individuals or species may also use aggregation or assort with similarly sized conspecifics as a protective mechanism against predation (e.g., Heupel and Simpfendorfer 2005; Holland et al. 1993) or to reduce competition with older, more competitive conspecifics, as has been shown in teleosts (e.g., Peuhkuri 1997). Additionally, sex segregation, commonly reported across elasmobranch species, can be shaped by both social and non‐social drivers. For example, maternal thermophily, driven by physiological needs of females, may produce aggregations based on surrounding water temperatures (e.g., Economakis & Lobel, 1998; Hight and Lowe 2007; Jirik & Lowe, 2012), while females of some species may aggregate to avoid male harassment (Klimley 1982). Furthermore, given that elasmobranchs have externally visible reproductive structures, these data are likely easier to acquire than for most fishes. Patterns of size and/or sex assortment, when analyzed, can therefore support further investigation into whether social mechanisms likely drive co‐occurrence, or if individuals are aggregating non‐socially.

#### 2.1.3 Evidence of Drivers of Co‐Occurrence

Identifying a common driver(s) is crucial to inferring the presence and nature of an elasmobranch aggregation (see McInturf et al. 2023). For social behaviours, individuals are attracted by the presence of conspecifics. In contrast, non‐social aggregations can be driven either by an environmental condition (e.g., optimal temperature) or resource (e.g., food). To this end, information on multiple drivers known to affect the distribution of a target species should be collected, depending on the source (e.g., fisheries or research). These data can be gathered in situ with each set or deployment, or retroactively (e.g., with satellite data), if in situ data are unavailable. For example, ocean temperatures at the depth of deployment can support whether individuals are aggregating in favourable thermal habitats. Stomach contents and prey type, if collectable, can indicate the presence of a foraging ground. Other species captured simultaneously might also serve as potential prey or imply a shared prey source. Ideally, these metrics are compared across multiple sites to determine if they predict species numbers and composition. If there is no relationship between environmental variables across sites where multiple individuals are consistently captured, it may suggest that other drivers, such as social attraction between individuals (Ward and Webster 2016), underpin group formation in these species. That hypothesis can then be more critically examined by considering the additional metrics proposed here.

#### 2.1.4 Identification of Specific Individuals

Discerning between potential non‐social aggregations and social groups is further strengthened by information on individual traits and identification of the individuals themselves. Social bonds and stable associations require that animals recognize and undergo repeated interactions with the same individuals (e.g., Bejder et al. 1998; Hinde 1976; Papastamatiou et al. 2022). The relationship between those individuals is founded on their history of interactions, from which preferences for associating with specific animals over time develop (Bejder et al. 1998; Hinde 1976; Papastamatiou et al. 2022). Elasmobranch social groups have been defined as specific or non‐specific, where individual identity does and does not matter, respectively (McInturf et al. 2023). Non‐specific social groups are often characterised by dynamic and weak associations (e.g., local enhancement in high quality habitat, schooling). In contrast, specific social groups, which require identification of specific individuals, are often formed by strong preferential associations creating clusters of conspecifics (e.g., Mourier et al. 2012) that can serve as the foundation for more complex social structures (e.g., dominance hierarchies, social communities; McInturf et al. 2023). Therefore, while phenotypic trait patterns (e.g., size, sex) can indicate whether individuals co‐occur due to the presence of others, individual identification is a stronger method of determining the degree of sociality they may exhibit. This information is unlikely to be reported in fisheries data for a few reasons. It is most often dependent on marking and recapturing individuals, which is rarely a priority. Furthermore, many fisheries are extractive, so seeing and identifying individuals on multiple occasions would be unusual. However, many research surveys include external, acoustic, or PIT (passive integrated transponder) tagging as part of their sampling procedure. For those that do not, there may be some instances in which identifying individuals is feasible without substantial additional effort (e.g., through photo‐ID), such as where individuals have unique external features (e.g., fin scarring, distinctive coloration, patterning; Anderson et al. 2011; Chin et al. 2015; Lonati et al. 2024).

#### 2.1.5 Identification of Kin Structure

Among other forms of individual identification, genetic data of captured individuals can offer unique insights into mechanisms of co‐occurrence. The formation of kin groups has been studied extensively in many animal societies; for instance, among mammals such as elephants (Wittemyer et al. 2009), dolphins (Wiszniewski et al. 2010), and bats (Carter and Wilkinson 2013; Kerth et al. 2011). Kin selection is a cornerstone in the evolution of social behavior and cooperation, where the benefits of kin structuring include inclusive fitness (Hamilton 1964) and reduced aggression (Olsén and JäUrvi 2005). However, these mechanisms are rarely studied in elasmobranchs (but see Guttridge et al. 2011; Mourier and Planes 2021; Newby et al. 2014). Catch data may be an optimal method of obtaining this information. Genetic samples are challenging to collect in cases where associations are measured via underwater observation (e.g., scuba surveys, underwater video recordings). However, these samples are much easier to access if the animals are captured. As with the tagging practices mentioned above, fisheries practices are unlikely to collect genetic data, but tissues could be obtained by researchers or observers on the vessel or later, from elasmobranchs retained onboard, to provide such information in the future. In comparison, collecting genetic data is frequently included in research survey protocols and may already be available for many datasets from these sources. Consistent capture of related individuals over time and space would warrant further examination to determine whether these occurrences are the product of spatial grouping (i.e., among neonates in a nursery area) or indicate evidence of social grouping.

### 2.2 Case Study: Juvenile Blacktip Reef Sharks

Here we illustrate the application of the framework proposed above with catch data collected on juvenile blacktip reef sharks ( *C. melanopterus* ) in Moorea, French Polynesia annually over a 10‐year period (2013–2023). Found on shallow reefs and sandflats throughout the Indo‐Pacific, adults of this species show a high degree of site attachment and spatial overlap and are known to aggregate, particularly when feeding (Papastamatiou et al. 2009; Mourier et al. 2012). Previous work on the adult population in Moorea has found communities of individuals created via nonrandom and temporally stable associations, driven at least in part by active social preferences (Mourier et al. 2012; Mourier and Planes 2021). However, as with most elasmobranchs, it is unknown whether neonates and juveniles exhibit the same social tendencies. Pups spend their first months in specific nursery areas, growing while sheltered from predators, with a very restricted home range through the first year (Bouyoucos, Romain, et al. 2020; Bouyoucos et al. 2022). As such, they could maintain connections with littermates and unrelated pups within the same nursery due to high spatiotemporal overlap, potentially promoting aggregation patterns.

We therefore first hypothesize that aggregation formation is a behavior that exists from early life stages in blacktip reef sharks (Hypothesis 1; see hypotheses and predictions associated with metric *i* in Table 1). In this case, we predict that multiple juveniles are captured together more frequently than single individuals. If so, we can then further explore an additional hypothesis. Specifically, given the social behaviors observed in adults of this species, we hypothesize that aggregations in juveniles are also formed by social grouping (Hypothesis 2a), and predict consistent evidence of conspecific co‐occurrence across time and sampling sites. This pattern will either support the presence of specific social groups (characterised by recaptures with the same individuals), non‐specific social groups (characterised by sex or size assortment or by high genetic relatedness; e.g., Guttridge et al. 2011), or specific social groups also characterized by assortment (see metrics *ii*, *iv*, and *v*; Table 1). Alternatively, juvenile blacktip reef shark aggregations may be non‐social (Hypothesis 2b). Here, we predict that environmental factors, such as habitat quality, significantly influence group formation, with variables like sampling site playing a strong role (metric *iii*; Table 1).

Given the challenges with determining a broadly applicable spatiotemporal scale at which to define aggregations in a specific species (McInturf et al. 2023), we test these hypotheses at two temporal levels to illustrate how the scale of examination influences study interpretation: across deployments and during instances of simultaneous captures. Importantly, the data used in this study were originally collected for population monitoring and physiological studies. As such, they represent a valuable opportunity for applying and testing our proposed framework on data acquired for purposes other than examining aggregations and their drivers.

### 2.3 Data Collection

All shark capture and research protocols were approved under arrêtés n° 9524, n° 5129, and n° 11,491 issued by the Ministère de la Promotion des Langues, de la Culture, de la Communication et de l'Environnement of the French Polynesian government and by the James Cook University Animal Ethics Committee (protocols A2089, A2394 and A2769). Data were collected over 10 consecutive parturition seasons (2013–2023) as part of long‐term, fisheries‐independent surveys carried out as a collaboration with the Centre de Recherches Insulaires et Observatoire de l'Environnement (CRIOBE) and the Physioshark Research Programme around Moorea, French Polynesia (17°30' S, 149°50' W). During these surveys, neonatal and juvenile blacktip reef sharks were caught using a 50 × 1.5 m gillnet with a 5‐cm mesh size set perpendicular to shore. Gillnets were set at dusk from ∼17:00 to 20:00 h at 10 sites (Apaura, Haapiti, Maharepa, Paorea, Papetoai, Pihaena, Tiki, Vaiane, Vaiare, and Valorie; Appendix S1) 5 days per week (i.e., Monday through Friday). These selected sites are established nursery areas nearly evenly spread out around the 60‐km coastline of Moorea, with each site sampled twice per month (e.g., Bouyoucos et al. 2022; Chin et al. 2015; Mourier et al. 2013; Mourier and Planes 2013). A majority of this sampling occurred between October and February, which represents the peak parturition season (Debaere et al. 2023; Mourier and Planes 2013), with some opportunistic data collected in September, March, and April.

For our analysis, we used only the deployments in which at least one shark was captured, and we removed any individuals whose sizes (~1 m) indicated that they may have matured to sub‐adulthood. We employed a “gambit‐of‐the‐group” approach (Whitehead & Dufault, 1999), considering sharks captured during the same deployment as co‐occurring in time and space. We analysed these data at two temporal scales to demonstrate the impact of sampling period on identifying potential instances of co‐occurrence (McInturf et al. 2023), as previous work on social networks has suggested that sampling scale and analytical decisions influence perceived patterns of association (e.g., Carter et al. 2015; Mourier et al., 2017b). First, data were grouped according to site, date, and set number (e.g., per net deployment) to simulate the information most often available from sources not explicitly aiming to address socioecological questions (e.g., fishing reports). In this research survey, however, specific capture time per individual was also reported. As such, we also examined the subset of cases in which individual juveniles were captured simultaneously.

For each deployment or simultaneous capture event, we determined the total number of sharks caught, their physical characteristics (i.e., variance in body size, the proportion and number of each sex caught), the identification of specific individuals (i.e., as determined through ID tags), and information that may indicate an external driver of co‐occurrence (i.e., location of capture). Additionally, we obtained genetic data on the same captured individuals used in this study from existing published analyses (Eustache 2024; Eustache et al. 2023, 2024) to assess kin‐based associations.

### 2.4 Analyses

To test our hypothesis that juvenile blacktip sharks aggregate, we identified and counted all instances of recaptured individuals, noting the fraction of cases in which they had been recaptured with the same sharks. Because there was evidence of some aggregation behavior (see “Results”), we then constructed two generalised additive models (GAMs) using the *mgcv* package in R (Wood 2011) to analyse data from the deployments where more than one shark was captured and simultaneous capture events (Table 2). GAMs were chosen for their flexibility, given our limited prior knowledge about the relationships between our response variables and predictors. Our first model treated the number of each sex caught per deployment or simultaneous capture event as a binomial response variable. The second model considered the variance in shark size (i.e., the difference between the maximum and minimum fork length; FL) per deployment or simultaneous capture event as a continuous response variable, which was square‐root transformed due to the right‐skewed distribution of values. In all models, the number of individuals captured and year were continuous, fixed‐effects predictors; month was a categorical fixed effect; and site was a random effect. Continuous predictors were smoothed using a thin‐plate regression, and weights were assigned based on the number of sharks per deployment. The performance of these models (hereafter referred to as “full models”) relative to null (intercept‐only) models was evaluated with a generalised likelihood ratio test, employing the anova.gam function (Wood 2011; R version 4.4.0).

**TABLE 2:** Model structure and results for generalised additive models employed in this study to examine potential assortment by juvenile blacktip reef sharks. Each model was used to examine patterns at two temporal scales: by deployment and instances of simultaneous capture. “S” indicates a smoothed term, whereas “bs = ‘re’” denotes a random effect. Significant predictors (*p* < 0.05) are shown in bold. All models except for model 2b (italicised) differed significantly from the null model in a likelihood ratio test (see text).

Scale of examination	Model structure	*R* ^*2*^
Deployment	1a. Sex ratio ~ **s(Total shark number)** + **s(Site, bs = “re”)** + **s(Year)** + **Month**	0.376
	2a. sqrt(Size variance) ~ **s(Total shark number)** + **s(Site, bs = “re”)** + s(Year) + **Month**	0.255
Simultaneous capture events	1b. Sex ratio ~ s (Total shark number) + s(Site, bs = “re”) + s(Year) + **Month**	0.137
	*2b. sqrt(Size variance) ~ s(Total shark number) + s(Site, bs = “re”) + s(Year) + Month*	*0.063*

## 3 Results

**FIGURE 1:**
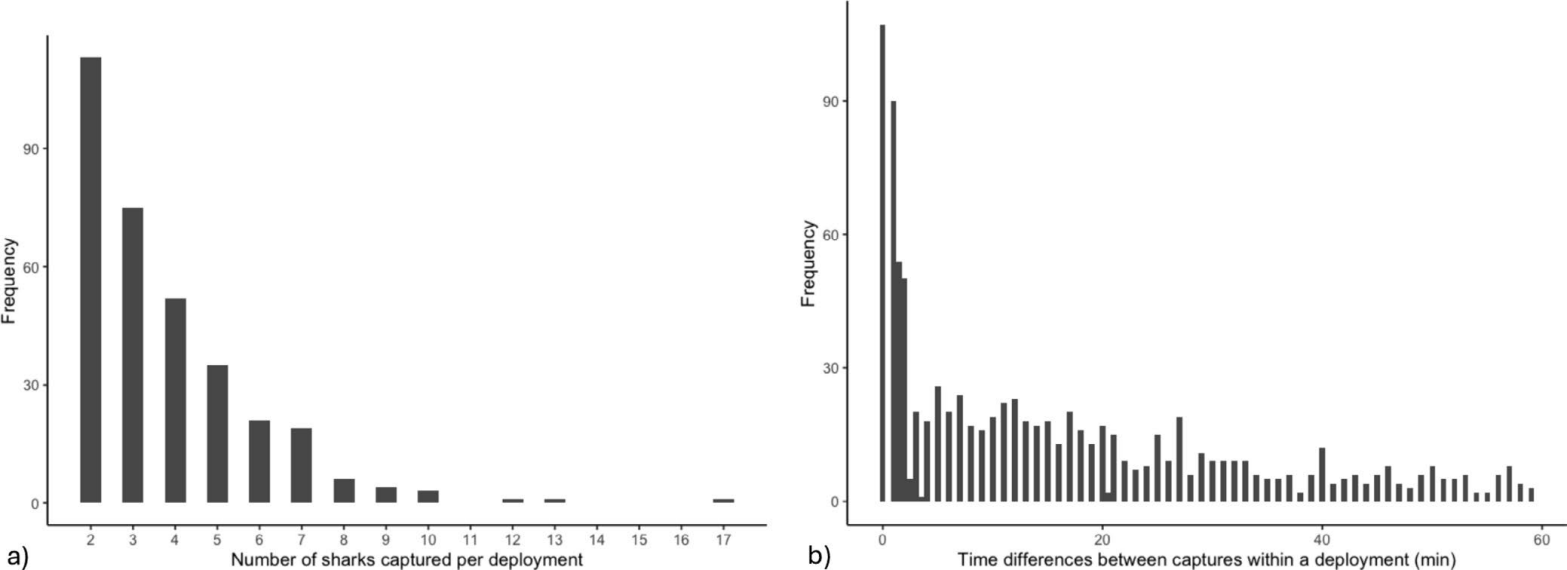
Juvenile blacktip reef shark catch data. (a) Number of sharks captured per deployment in Moorea between 2013 and 2023. (b) Time differences between individual shark captures. Data were only included for deployments in which more than one shark was captured.

A total of 502 net deployments resulted in the capture of 1420 juvenile blacktip reef sharks for which sex was determined (629 female, 850 male). Though sharks were captured across all months of the sampling period (September through April), most (~94%) were caught between October and February. Of all deployments, a majority (66%, *n* = 344) involved the capture of more than one shark. Single shark captures accounted for approximately 34% of all instances observed (*n* = 176). In instances of multi‐shark captures per deployment, most involved just two sharks, with six instances where 10 or more sharks were captured (maximum: 17 sharks; Figure 1a). Excluding one instance of a night fishing pilot study (> 20 h, 7 sharks captured), the time interval between the first and last shark captured in each deployment ranged from 0 (i.e., indicating simultaneous capture) to 3 h 36 min, with a median time of 25 min. Within a given deployment, capture time between individuals varied from 0 to 59 min (median: 10 min) (Figure 1b). There were also 101 instances of simultaneous captures between at least two individual juveniles (range: 2–7; median: 2).

Upon analysing instances where sharks were captured and tagged in the same net and were subsequently recaptured together, we identified a total of 205 recapture events. Among these, only 19 cases involved sharks being captured alongside the same individual with which they were initially captured. Genetic analysis found that, in cases where data were available (prior to 2021), no individuals recaptured together were identifiable as siblings. However, all individuals were recaptured at the same sites where their initial capture occurred (Appendix S2).

**FIGURE 2:**
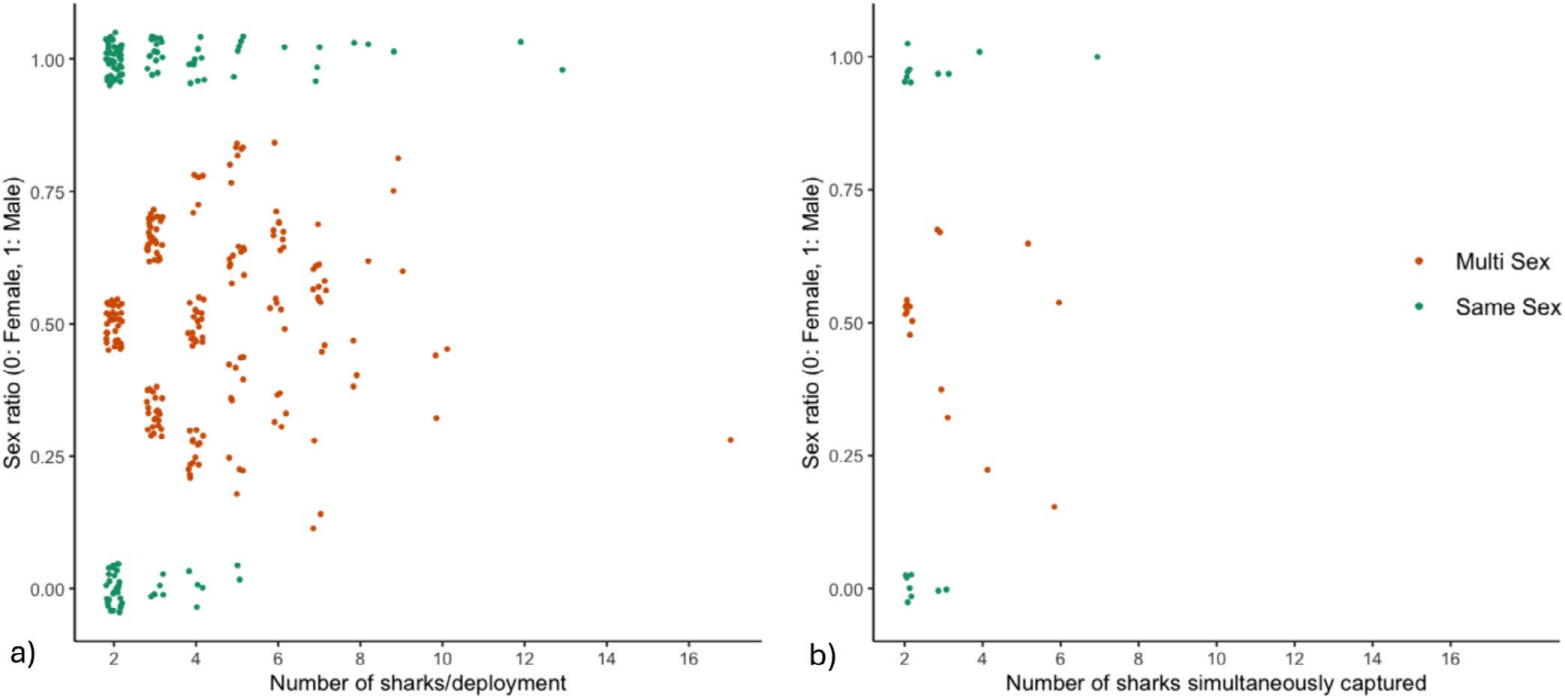
Sex ratio in juvenile blacktip reef sharks. Relationship between sex ratio and (a) number of sharks per deployment for all deployments in which at least two sharks were captured, and (b) number of sharks simultaneously captured. Sex ratio ranges from 0 (all females) to 1 (all males), and colours indicate multi‐sex (orange) or same sex (green) co‐occurring individuals.

To determine whether sharks might be assorting by traits rather than individual identity or kinship, we analysed the sex and size distributions of deployments and simultaneous capture events in which more than one shark was caught. Among deployments, 203 (40%) resulted in the capture of both sexes, while there were 188 all‐male catches and 111 all‐female catches (Figure 2a). A similar pattern was evident when we examined sharks that had been captured together instantaneously (Figure 2b). Both males and females were captured in 46% of these instances, while there were only 19 all‐female and 36 all‐male captures. Additionally, we observed that both males and females were almost equally likely (46% and 48%, respectively) to be captured alone as in any group.

**FIGURE 3:**
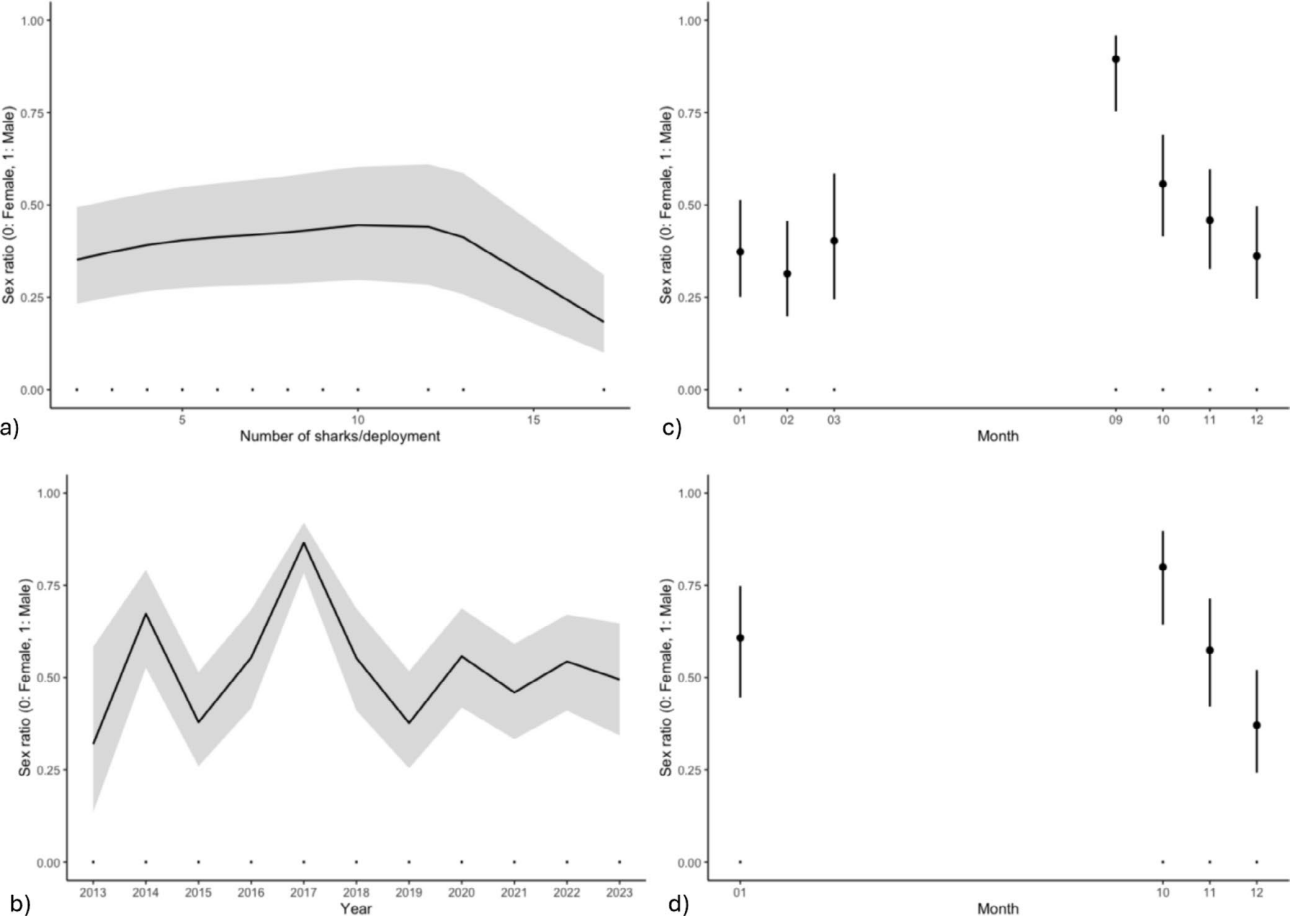
Partial response plots from a GAM built using data at the deployment scale (a–c) and for simultaneous capture instances (d). Only statistically significant relationships are depicted. At the deployment scale, these include (a) sex ratio and number of sharks captured per deployment, and variation in sex ratio (b) by year and (c) throughout the sampling season among deployments. For simultaneous captures, (d) month was a significant predictor. Monthly data are shown for all months that sampling occurred and for which sufficient data were available (see text for details).

A likelihood ratio test comparing our null (intercept‐only) model and the full model at the deployment scale (Model 1a; Table 2) revealed a statistically significant difference in deviance explained (deviance = 1037.1; degrees of freedom = 28.5; *p* < 0.05). The full model (R^2^ = 0.376) included the number of sharks per deployment and year as continuous fixed effects, month as a categorical fixed effect, and site as a random effect. Data from April were removed due to an insufficient sample size (i.e., 3 deployments total). This model indicated that the sex ratio was significantly influenced by the total number of sharks per deployment (edf = 3.45; Chi‐square = 59.2; *p* < 0.05). Specifically, the sex ratio skewed toward females at lower numbers, shifted toward an equal proportion as the number of sharks captured per net increased, and then again became female‐dominated at the highest number (Figure 3a). We also ran this model without an outlying datapoint, where the largest mixed‐sex catch was disproportionately female, to determine whether our results were robust. Doing so resulted in the opposite pattern, such that the sex ratio became male‐skewed with an increase in the number of sharks captured (Appendix S3). Year (edf = 8.81; Chi‐square = 276.42; *p* < 0.05) and specific months (September [*z* = 6.01; *p* < 0.05], October [*z* = 6.70; *p* < 0.05], November [*z* = 3.67; *p* < 0.05]) were also significant predictors, such that the sex ratio varied annually (with peaks in male‐dominated captures in 2014 and 2017, and female captures in 2015 and 2019; Figure 3b) and a greater proportion of males were represented in the early part of the season (September, October, November; Figure 3c). Finally, site was also a statistically significant random effect in this model (edf = 8.08; Chi‐square = 138.0; *p* < 0.05).

In cases where individuals were captured simultaneously (Models 1b, 2b; Table 2), there were fewer observations in February, March, April, and September compared to our examination at the deployment scale (< 5 instances of simultaneous capture each month). As such, these months were excluded from this component of our analysis. When examining sex ratio patterns within these data (Model 1b), we found a significant difference in deviance explained between our full and null models (deviance = 71.88; degrees of freedom = 10.86; *p* < 0.05). As above, specific months (October [*z* = 2.59; *p* = 0.009], December [*z* = −3.51; *p* = 0.004]) were significant predictors in this full model (Table 2). Specifically, the sex ratio skewed toward males in October and then shifted toward females in December (Figure 3d). However, site, year, and total number of sharks were not significant, and explanatory power decreased (*R* ^2^ = 0.137) for this model compared to the deployment model.

**FIGURE 4:**
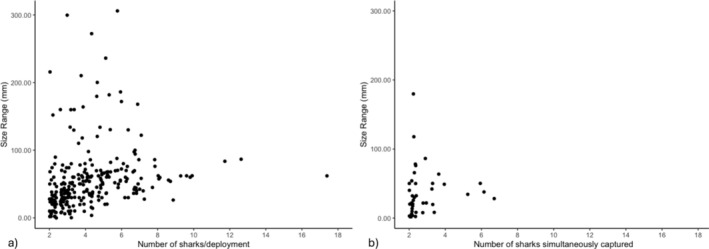
Number and size range of juvenile blacktip reef sharks. Relationship between variance in fork length (the difference between maximum and minimum sizes, in mm) and the number of juvenile blacktip reef sharks for (a) all deployments in which at least two sharks were captured and (b) instances of simultaneous capture.

We also examined the effect of these same predictors on size variation at the deployment and simultaneous capture scales (Model 2a, b; Table 2). Individual sizes in our dataset varied widely, from 392 to 792 mm fork length. We observed no clear difference in size range between sexes; female lengths spanned from 392 to 792 mm, while male lengths ranged from 398 to 750 mm. Within a given deployment, the size differences between captured sharks ranged from 0 to 306 mm (Figure 4a). Among simultaneously captured individuals, there was less variation, with size differences from 0 to 180 mm (Figure 4b).

**FIGURE 5:**
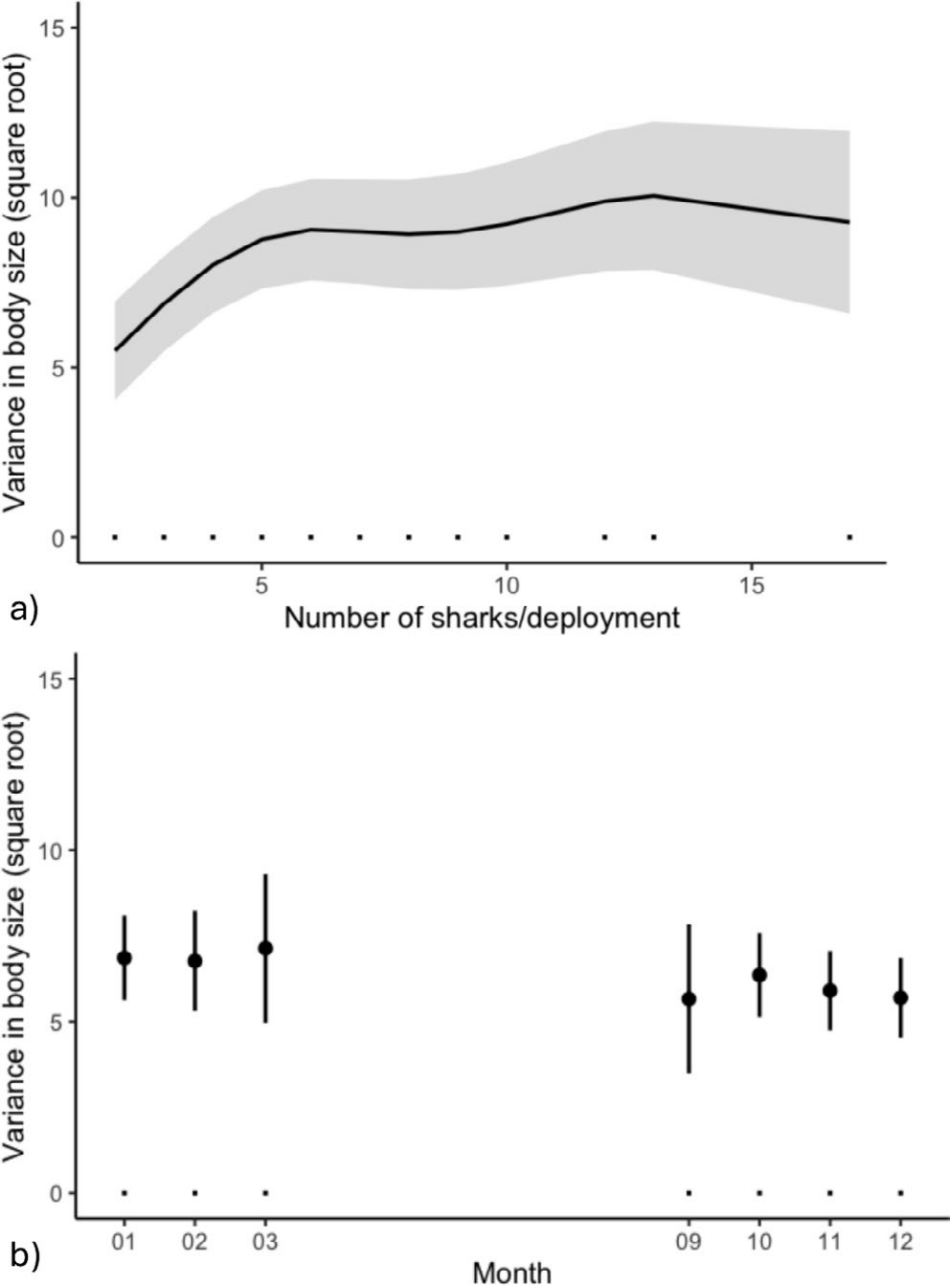
Partial response plots from a GAM built using data at the deployment scale. Only statistically significant relationships are depicted. These include (a) between body size variance (calculated as the difference in maximum and minimum length of sharks captured, in mm) and number of sharks captured per deployment, and (b) size variance throughout the sampling season among deployments. Monthly data are only shown for months that sampling occurred.

A likelihood ratio test comparing our full model and null (intercept‐only) model revealed a statistically significant difference in deviance explained (*p* < 0.05) for only the deployment scale (deviance = 2798.3; degrees of freedom = 24.419; *p* < 0.05), but not for simultaneous capture instances. Among deployments, the full model (*R* ^2^ = 0.255) predicted that the number of sharks had a significant impact on the variance in size (edf = 5.30; *F* = 18.41; *p* < 0.05). This variance initially increased but then stabilized as the number of sharks per deployment increased (Figure 5a). Of the other predictors, year was not significant, but the random effect of site (edf = 4.51; *F* = 1.17; *p* = 0.17) and fixed effect of month (Figure 5b) influenced size variation, with higher variance in specific months (i.e., November [*t* = −2.28, *p* = 0.02], December [*t* = −2.76, *p* = 0.01]).

## 4 Discussion

Our framework is designed to leverage existing elasmobranch catch data through the lens of socioecology. Quantifying behavioural interactions, interindividual social dynamics, and the drivers of group formation in such highly mobile marine organisms remains a significant logistical challenge. In response to this challenge, our proposed framework employs existing catch data to elucidate patterns of aggregation by examining metrics related to individual capture, phenotypic traits, and potential social dynamics. Each metric within our framework targets a specific aspect necessary to identify an elasmobranch aggregation and infer the extent to which they result from social and/or environmental factors (McInturf et al. 2023). Utilising our case study as an example, we critically examined the capabilities and limitations of this framework. In doing so, we showed that blacktip reef sharks during early life stages do tend to aggregate (Table 3), as has been documented in adults (Mourier et al. 2012; Mourier and Planes 2021). However, unlike in adults, the drivers of these aggregations in early life are likely to be primarily environmental rather than social (Table 3). From this exercise, we will highlight how future data collection can be optimized to enhance the behavioural insights derived from various capture methods.

**TABLE 3:** Application of the proposed framework to address hypotheses outlined in our case study. These metrics are used to determine whether aggregations may be occurring in juvenile blacktip reef sharks in Moorea, and if so, what type.

Descriptive metric	Finding from this study	Supports aggregation (H1)	Supports non‐social aggregation (H2a)	Support social grouping (H2b)
Number and density of individuals captured	Most deployments (66%) captured multiple sharks.	Yes	—	—
Drivers of co‐occurrence (site location)	All recaptured individuals that had been captured together were found at their original capture locations, and site was a significant predictor in deployment‐scale models.	—	Yes	No
Individual phenotypic traits (size/sex)	There was a broad range of sizes among sharks captured per deployment, and 60% of deployments were single‐sex catches.	—	Yes	No
Identification of individuals	Less than 10% of captured individuals were recaptured together.	—	Yes	No
Identification of kin structure	There were no apparent genetic linkages between recaptured individuals that had initially been captured together.	—	Yes	No

### 4.1 Case Study: Juvenile Blacktip Reef Sharks

The catch data on juvenile blacktip reef sharks that we collected over a decade in Moorea, French Polynesia, originated primarily from a long‐term scientific monitoring program. Though not specifically designed for behavioural analyses, these data provided comprehensive information on shark traits, deployment location and duration, and specific times of individual shark capture. As such, they enabled us to thoroughly examine each aspect of our framework for investigating patterns and drivers of aggregations, while assessing how robust such patterns may be to analytical decisions such as the temporal scale of analysis. Given the well‐documented social behaviour of adult blacktip reef sharks within this system (Mourier et al. 2012; Mourier and Planes 2021), the insights gained from studying juveniles offer a valuable comparative perspective, offering potential to elucidate the ontogenetic development of social behaviours.

Blacktip reef sharks are a prevalent species in Indo‐Pacific coral reefs (Compagno et al. 2005; Mourier et al. 2012), with adults often observed in small aggregations (Mourier et al. 2012; Papastamatiou et al. 2009). Our data, where most deployments contained multiple sharks, support the notion that aggregations are also common among juvenile blacktip reef sharks in this region (Hypothesis 1; Table 2). This was unsurprising, as sampling took place in known nursery areas where individuals at early life stages occupy a small home range (Bouyoucos, Romain, et al. 2020; Bouyoucos et al. 2022) until they are large enough to disperse. As such, this finding contributes to a growing body of existing evidence suggesting that nurseries function as aggregation sites for juvenile blacktip reef sharks (e.g., Weideli et al. 2019; Bouyoucos, Romain, et al. 2020; Bouyoucos and Rummer 2020; Bouyoucos, Watson, et al. 2020; Bouyoucos et al. 2022).

A key next step in our analysis was to determine whether there were underlying structures among conspecifics within these aggregation areas (Hypothesis 2). To do so, we analysed site location, individual identities, and phenotypic traits of the captured individuals. Previous research on adult blacktip reef sharks suggests that aggregations may be formed by stable communities, influenced in part by the active choice of individuals to associate with one another (Mourier et al. 2012; Mourier and Planes 2021). Consequently, we initially investigated the presence of specific social grouping, which would indicate that individuals can recognise and preferentially associate with certain conspecifics. However, our findings provided limited support for this type of aggregation. Tagging data revealed 205 recaptures during the 10‐year study period, but among these, only 19 instances involved individuals being recaptured together. This pattern could be due in part to high early life‐stage mortality, such that individuals do not survive long enough to be recaptured. Nonetheless, it contrasts with observations in adult populations, where individuals were often sighted repeatedly, sometimes more than four times, across various study sites (Mourier et al. 2012). Based on these observations, a compelling hypothesis arising from our study is that the ability for individual recognition and the formation of stable social groups may develop ontogenetically in blacktip reef sharks.

We also investigated the potential for non‐specific social grouping in juvenile blacktip reef sharks, focusing on trait‐based assortment. Class segregation, commonly reported among many elasmobranch species, may be particularly significant for juvenile sharks, as it is thought to offer antipredator advantages (e.g., Guttridge et al. 2011; Krause 1994). For this study, we selected sex and length as the primary phenotypical traits of interest, as previous research has demonstrated that these influence assortment in adult blacktip reef shark communities (Mourier et al. 2012). However, our results suggest that trait‐based assortment may not be the primary driver of aggregations in juvenile blacktip reef sharks (Table 2). Approximately 60% of our deployments comprised single‐sex catches, which aligns with existing evidence suggesting that mixed‐sex communities of adults are common in this system (Mourier et al. 2012). However, our full model on the deployment scale did suggest a significant relationship between the number of sharks per deployment and sex ratio. Predictions from this model showed a slight skew from female‐dominated captures toward equal sex proportions as the number of sharks per deployment increased (Figure 3a). Yet we found that this relationship was likely driven by an outlying data point (Figure 2a; Appendix S3), suggesting that more data are needed to robustly assess sex ratio patterns when large numbers of individuals are captured. Conversely, our model built using data from simultaneous shark captures showed no statistically significant relationship between sex ratio and the number of individuals caught together, indicating that this correlation is not robust to the scale of examination. Additionally, we observed that males and females were nearly equally likely to be captured either alone or together, although there were some notable sex differences in the number of animals captured in single‐sex catches. The largest deployments comprised of a single sex (up to 14 individuals) were all male, while all‐female deployments never exceeded five sharks. For comparison, the largest mixed‐sex deployments reached up to 17 individuals. Similar trends were observed in instances of simultaneous capture (with a maximum of 7 sharks per all‐male catch and 3 per all‐female). Thus, although mixed‐sex captures were prevalent in our data, there may also be some indication that juveniles exhibit similar sex variation in associations as reported in adult populations, where males have been observed to be more gregarious than females (Mourier and Planes 2021). Yet in general, given that the significance of these results varies by scale and the data indicate no overwhelming support for sex assortment, our framework underscores the need for continuing to assess sex variation in social behaviour ontogenically to better understand the evolution of social dynamics in elasmobranch species.

Our analysis of size variance within deployments and simultaneous captures also yielded complex results. We noted a broad range in sizes among individuals, regardless of the number of sharks captured per deployment. Yet our deployment‐scale model revealed that size variance tended to plateau as the number of sharks captured increased. This observation suggests potential limits on the sizes of individuals that co‐occur, although the underlying mechanisms remain unclear. Several factors could contribute to this pattern: it might reflect individual choices within the aggregations or be a consequence of the limitations imposed by the mesh size of the nets used for capture. It is also likely that we captured a transition period in ontogenetic space use, as larger individuals start to disperse from the nursery location. However, when we examined patterns of co‐occurrence based on simultaneous capture events, our model was not significantly different from the intercept‐only model. This indicates that deliberate size assortment among individuals is likely not the mechanism driving the results observed in the deployment model. Rather, as all captured animals were within the same general age class, size variation at a finer scale (i.e., within a given class) may not lead to assortment. Alternatively, previous studies on this population (i.e., Weideli et al. 2019) have suggested that annual growth rates of juvenile blacktip reef sharks are highly variable, perhaps due to limited prey availability, and such heterogeneity in growth among individuals could reduce the likelihood of assortment in this age class. By adulthood, size and growth are more stable and homogenous, which is also when size assortment has been documented in this species (Mourier et al. 2012). While our study does not pinpoint the exact mechanisms driving size variation, it highlights an important area for future research.

Littermates among blacktip reef sharks have been shown to share the same nursery sites (Eustache et al. 2023), providing optimal conditions for kin‐based associations to arise. Yet we found no evidence of genetic linkages among any recaptured individuals for which we had data, confirming that these communal nurseries are shared by multiple litters. This was also notable, given that previous studies of this population have determined that individuals initially captured together are occasionally related, potentially suggesting companionship behavior during early weeks of life (Debaere et al. 2023; Eustache et al. 2023). However, our results align more with social network analyses on adults in this area, which have found that kinship does not underlie patterns of co‐occurrence (Mourier and Planes 2021). Kin associations are prevalent in many social organisms, such as mammals in both terrestrial (elephants, Archie et al. 2005; meerkats, Clutton‐Brock et al., 2001) and marine (killer whales, Ford 2019; bottlenose dolphins, Wiszniewski et al. 2009) environments. Kin associations appear less common in sharks, likely due to the absence of parental care, often a key factor in promoting extended interaction among offspring after birth (Pratt and Carrier 2001). Sharks typically leave their progeny in specific nurseries separate from adult habitats (Mourier and Planes 2013, 2021). The lack of genetic relatedness structure among adults could be due to the high likelihood that juveniles do not leave their nursery ground with other kin and because the small litter size in this species, combined with high early‐stage mortality, does not promote the conditions for kin to survive to adulthood (Mourier and Planes 2021). Our results further suggest that there is ephemeral kin assortment even within the nursery ground and also suggest that this could be partly due to small litter size. Blacktip reef sharks typically have few young, with a maximum of five pups (Mourier et al. 2013) and only 3–5 in Moorea (Eustache et al. 2023). Thus, the likelihood of encountering kin in nursery habitats may be reduced after an initial companionship period due to natural mortality and as individuals grow and disperse within the broader area (Guttridge et al. 2011). This contrasts with the more complex kin‐based social structures observed in some terrestrial and marine mammal species, where larger group sizes and prolonged parental care facilitate kin interactions (Clutton‐Brock 2016).

Ultimately, the lack of apparent structure by individual, relatedness, size, or sex in juvenile blacktip reef shark captures indicates little support for social affiliation within aggregation sites in Moorea (Hypothesis 2a). Rather, our analysis supports an alternate hypothesis that aggregations at this life stage are evident but non‐social (Hypothesis 2b). All recaptured individuals were found at their original capture locations, and our statistical models identified the capture site as a significant predictor of both sex ratio and size variation among the sharks at the deployment scale (Table 2). This is supported by previous work showing that juveniles are generally confined to their nursery areas, and their small home range and low mobility (Bouyoucos, Romain, et al. 2020; Bouyoucos et al. 2022, 2024) can lead to proximity among conspecifics. Temporal variables such as month were also statistically significant in nearly all models, and year was a significant predictor of sex ratio for the deployment models. Although recent studies in Moorea suggest that juvenile and neonate blacktip reef sharks are resilient to variation in certain abiotic conditions (i.e., temperature, oxygen, pH, salinity, lunar phase, depth; Bouyoucos et al. 2024; Eustache et al. 2024), our findings strongly suggest that external seasonal and annual factors, perhaps related to reproductive philopatry, resource availability, dispersal ability, natural mortality, and/or habitat quality (e.g., Bouyoucos et al. 2022, 2024), are influential in driving patterns of co‐occurrence. To some degree, this aligns with existing work on adult blacktip reef sharks, which indicates that stable associations within shark communities are spatially constrained, influenced by non‐social mechanisms (e.g., resource availability) as well as social drivers (Mourier et al. 2012). Thus, our collective insights imply that nursery habitats play a crucial role in shaping early population dynamics and spatial behavior by enabling the formation of non‐social aggregations, which later manifest as spatially constrained, non‐random relationships in adults.

### 4.2 Caveats and Future Directions

Here, we have demonstrated the utility of catch data in identifying aggregations and assessing whether they are likely to be driven by social or non‐social mechanisms. While our case study illustrates the application and value of our proposed framework, there are several caveats that may influence its broad applicability to various species or environments. Such caveats should be considered when interpreting catch data to identify systems where further socioecological work may be warranted. For example, our case study was based on uniquely rich data from extensive and repeated research surveys in the same system. As such, they provided an ideal suite of metrics for our framework, including genetic information, fine‐scale capture timing, and individual identification. The longitudinal nature of these data, covering the same sites over multiple years, enabled us to track recaptures and evaluate spatial patterns over time. Furthermore, they also allowed us to determine that our analytical outcomes, as guided by the proposed framework, were not always robust to the temporal scale of examination. Yet catch data from other sources may lack relevant information for addressing questions pertaining to elasmobranch aggregations. Future efforts should therefore encourage those involved in catch data collection to slightly modify their protocols to improve the socioecological value of these data. For example, while most fisheries are unlikely to maintain tagging or tissue sampling programs, the presence of observers on vessels could enhance data collection on individuals captured. Additional adjustments could range in effort and priority depending on feasibility and fishing goals, including recording size/sex, collecting genetic samples, obtaining photographs, visually tagging specific species (e.g., with Floy tags), and reporting variables such as mesh size or line type, the duration of deployment for lines or nets, spatial distances covered by the fishing effort, and the time of individual shark capture in certain circumstances.

Additionally, this framework is inherently influenced by biases introduced by fishing activity, including gear type and effort. Our case study data were derived from gillnets that were consistently observable and were not accompanied by an attractant. However, as the goal of fishing is often to maximise the number of individuals captured, many fishing techniques (e.g., longlines) use luring devices or strategies (e.g., bait, light) that may artificially generate capture patterns indicative of an aggregation. Similarly, techniques that are less discerning in target catch (e.g., trawls) may capture individuals over larger spatial and temporal scales than are feasible to assume they would be aggregating. Moreover, fisheries or surveys may more easily cover specific habitats (e.g., reef, coastal) than others (e.g., deep‐sea, pelagic), and as such, our framework may be more readily applied to species within more accessible systems at present, particularly when considering the likelihood of mark‐recapture and obtaining genetic samples. We also assumed in this study that individuals survived capture, such that examining patterns of recapture would be feasible, but different species exhibit varying physiological responses to stress that are also likely dependent on fishing technique and gear type (Bouyoucos et al. 2018).

Nonetheless, as we have demonstrated, this framework can be incredibly valuable as a tool for obtaining behavioural knowledge on organisms that are often challenging to study. As such, and given these caveats, we have provided guidelines in our framework for obtaining sufficient supporting evidence, beyond the number or density of individuals captured, that can be used to justify further exploration into socioecological behaviours of different species. However, we also advocate for examining data from multiple gear types when available, and for determining the feasibility of inferring information from different sources based on a priori knowledge of the study system. Regardless of how much information is available, thorough reporting of assumptions and context is essential for future work employing this approach.

### 4.3 Concluding Remarks

The proposed framework to leverage catch data can broaden our knowledge of elasmobranch aggregations and social behaviors. Beyond the clear applications in socioecological research, the insights gained from the catch data analysed through this framework can also inform conservation strategies. Elasmobranchs, as one of the most vulnerable classes of vertebrates, face significant population declines due to their K‐selected life history strategies (Conrath and Musick 2012; Ward‐Paige et al. 2012). Their role as apex or meso‐predators underscores the ecological impact of their loss (Dedman et al. 2024; Hammerschlag et al. 2019; Wheeler et al. 2020). With populations already declining worldwide (e.g., Dulvy et al. 2021; Pacoureau et al. 2021; Ward‐Paige et al. 2012), common threats including overfishing, incidental capture, habitat destruction, and climate change (Jennings et al. 2008; Mandelman et al. 2013; Ward‐Paige et al. 2012; Wheeler et al. 2020) are often exacerbated by aggregation behaviors (McInturf et al. 2023). Furthermore, spatial management often protects elasmobranch species, particularly those aggregating in “hotspots” (Hazen et al. 2013; Myers et al. 2000). However, the impact of intraspecific associations on spatial population structuring remains underexplored (Mourier et al. 2012). Thus, catch data not only shed light on species distribution patterns but also offer insights into how conspecific associations affect habitat use and population dynamics, addressing an urgent need in elasmobranch management (McInturf et al. 2023).

## Author Contributions

**A. G. McInturf:** conceptualization (lead), formal analysis (lead), methodology (lead), project administration (lead), visualization (lead), writing – original draft (lead), writing – review and editing (lead). **M. Cantor:** conceptualization (supporting), formal analysis (supporting), investigation (supporting), methodology (supporting), writing – review and editing (supporting). **I. A. Bouyoucos:** conceptualization (supporting), data curation (equal), investigation (supporting), methodology (supporting), writing – review and editing (supporting). **T. K. Chapple:** investigation (supporting), methodology (supporting), resources (supporting), supervision (supporting), writing – review and editing (supporting). **S. F. Debaere:** conceptualization (supporting), data curation (equal), investigation (supporting), methodology (supporting), writing – review and editing (supporting). **K. Eustache:** data curation (equal), formal analysis (supporting), methodology (supporting), writing – review and editing (supporting). **J. Mourier:** formal analysis (supporting), investigation (supporting), methodology (supporting), writing – review and editing (supporting). **S. Planes:** data curation (equal), formal analysis (supporting), investigation (supporting), methodology (supporting), writing – review and editing (supporting). **J. A. Sulikowski:** resources (supporting), supervision (supporting), writing – review and editing (supporting). **N. A. Fangue:** conceptualization (supporting), methodology (supporting), supervision (supporting), writing – review and editing (supporting). **K. W. Zillig:** conceptualization (supporting), investigation (supporting), methodology (supporting), writing – review and editing (supporting). **J. L. Rummer:** conceptualization (equal), data curation (supporting), investigation (supporting), methodology (supporting), project administration (supporting), supervision (supporting), writing – original draft (supporting), writing – review and editing (supporting).

## Conflicts of Interest

The authors declare no conflicts of interest.

## Supporting information


Appendix S1.

## Data Availability

Data presented in this manuscript are available in a figshare repository: https://doi.org/10.6084/m9.figshare.28603916.v1.
