# Supplementary material for: Catch Data Can Unravel Elasmobranch Aggregation Dynamics and Group Behaviours
Source: Ecol Evol. 2025 Mar 30;15(4):e71107. doi: 10.1002/ece3.71107 (PMC11955303; doi:10.1002/ece3.71107)
Supplement: Supplementary file 1 — Appendix S1. [file ECE3-15-e71107-s001.docx]

**Appendix 1:**

***A map of juvenile blacktip reef shark capture sites around Moorea, French Polynesia (17° 30’ S, 149° 50’ W).*** *The Centre de Recherches Insulaires et Observatoire de l’Environnement (CRIOBE)* *is also listed (figure from Bouyoucos et al., 2022, used with permission).*

**
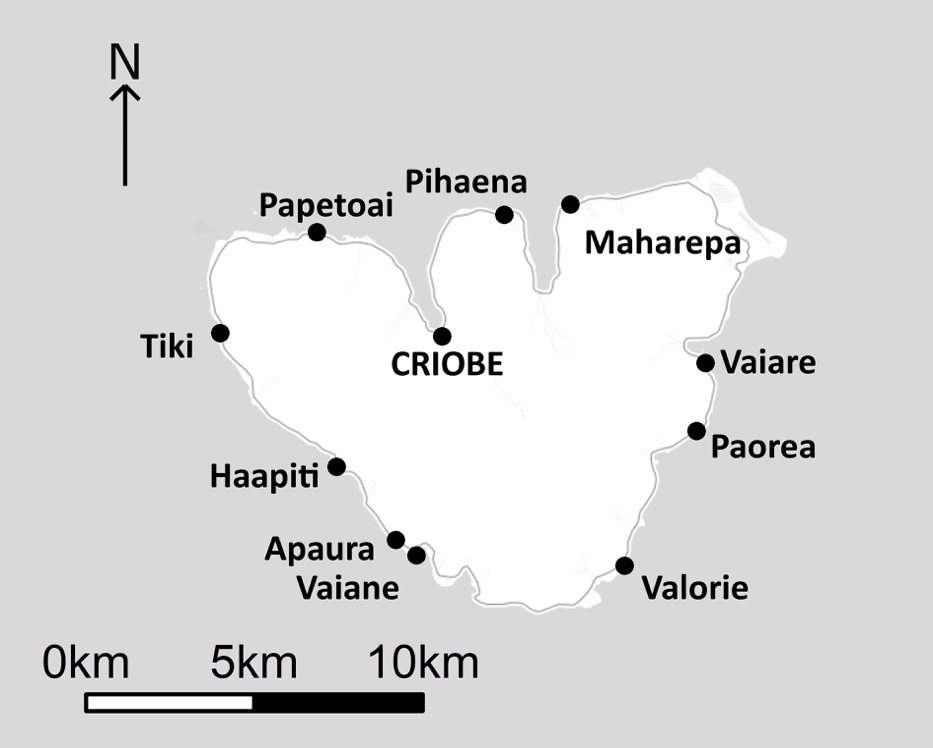
**

**Appendix 2:**

***Instances of tagged juvenile blacktip reef sharks that were recaptured with the same individuals****. These are categorized by field season (2016-2023; no recaptures occurred in field seasons prior to this time), dates of capture/recapture, and site. The total number of recaptures by field season (inclusive of individuals recaptured together) are listed under the field season years.*

| Field seasons |  | Tag numbers of sharks recaptured together | Dates (captured; recaptured) | Site |
| --- | --- | --- | --- | --- |
| 2016-2017  (Total recaptures: 24) | a | COFA19745, COFA19746 | Dec 22, 2016; Jan 17, 2017 | Haapiti |
|  | b | 955000004245503; 955000004245511 | Oct 26, 2017; Nov 13, 2017 | Paorea |
| 2017-2018  (Total recaptures: 25) | c | 955000004245264; 955000004245285 | Nov 6, 2017; Dec 7, 2017 | Vaiare |
|  | d | 955000004245101, 955000004245130, 955000004245498, 955000004245153 | Dec 6, 2017; Dec 22, 2017 | Paorea |
|  | e | 955000004244969, 955000004245487 | Jan 17, 2018; Jan 26, 2018 | Paorea |
| 2018-2019  (Total recaptures: 18) | f | 955000004245075, 955000004245103 | Dec 10, 2018; Jan 22, 2019 | Paorea |
| 2019-2020  (Total recaptures: 19) | g | 955000004245027, 955000004244954 | Oct 28, 2019; Nov 25, 2019 | Maharepa |
|  | h | 955000004245178, 955000004245282 | Oct 28, 2019; Dec 4, 2019 | Maharepa |
|  | i | 955000004245384, 955000004245316 | No 20, 2019; Dec 9, 2019 | Vaiare |
|  | j | 955000004245364, 955000004245333 | Dec 4, 2019; Dec 12, 2019 | Maharepa |
| 2020-2021  (Total recaptures: 31) | k | 955000005031980, 955000005032128, 955000005031947, 955000005031935 | Oct 6, 2020; Dec 22, 2020 | Vaiare |
|  | l | 955000005032017, 955000005032035 | Dec 7, 2020; Dec 21, 2020 | Vaiare |
|  | m | 955000005031816, 955000005031854 | Jan 8, 2021; Jan 24, 2021 | Valorie |
| 2021-2022  (Total recaptures: 43) | n | 955000004999337, 955000004999446 | Nov 30, 2021; Dec 14, 2021 | Paorea |
|  | o | 955000004999445, 955000004999454 | Nov 30, 2021; Dec 28, 2021 | Paorea |
|  | p | 955000004999454, 955000004999386 | Nov 30, 2021; Jan 25, 2022 | Paorea |
| 2022-2023  (Total recaptures: 45) | q | 955000004999437, 955000004999279 | Jan 11, 2022 (season prior), Sept 8, 2022 | Paorea |
|  | r | 955000004999505, 955000004999526 | Sept 30, 2022; Oct 7, 2022 | Papetoai |
|  | s | 955000004999659, 955000004999634 | Nov 28, 2022; Dec 29, 2022 | Vaiare |

**Appendix 3:**

**
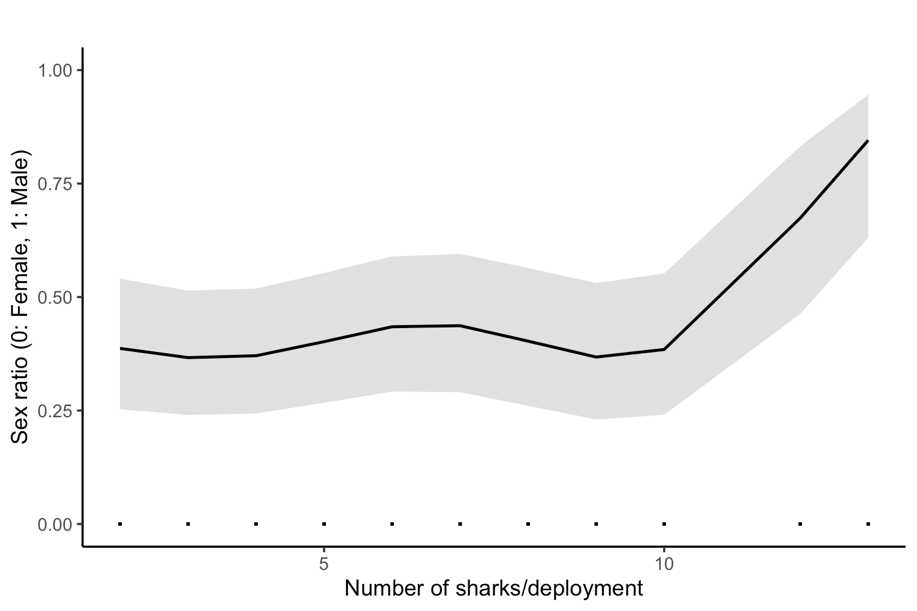
**

***Partial response plot from a GAM built using data at the deployment scale depicting the significant relationship between sex ratio and number of sharks captured per deployment****. The outlying datapoint for the largest mixed-sex deployment is omitted here.*
